# When fast is better: protein folding fundamentals and mechanisms from ultrafast approaches

**DOI:** 10.1042/BCJ20160107

**Published:** 2016-08-30

**Authors:** Victor Muñoz, Michele Cerminara

**Affiliations:** * National Biotechnology Center, CSIC, Darwin, 3. E-28049 Madrid, Spain; † IMDEA Nanosciences Institute, Calle Faraday 9, E-28049 Madrid, Spain; ‡ School of Engineering, University of California Merced, 5200 N. Lake Road, Merced, CA 95343, U.S.A.

**Keywords:** conformational rheostats, molecular dynamics simulations, nuclear magnetic resonance, protein folding energy landscapes, single-molecule spectroscopy, ultrafast kinetic methods

## Abstract

Protein folding research stalled for decades because conventional experiments indicated that proteins fold slowly and in single strokes, whereas theory predicted a complex interplay between dynamics and energetics resulting in myriad microscopic pathways. Ultrafast kinetic methods turned the field upside down by providing the means to probe fundamental aspects of folding, test theoretical predictions and benchmark simulations. Accordingly, experimentalists could measure the timescales for all relevant folding motions, determine the folding speed limit and confirm that folding barriers are entropic bottlenecks. Moreover, a catalogue of proteins that fold extremely fast (microseconds) could be identified. Such fast-folding proteins cross shallow free energy barriers or fold downhill, and thus unfold with minimal co-operativity (gradually). A new generation of thermodynamic methods has exploited this property to map folding landscapes, interaction networks and mechanisms at nearly atomic resolution. In parallel, modern molecular dynamics simulations have finally reached the timescales required to watch fast-folding proteins fold and unfold *in silico*. All of these findings have buttressed the fundamentals of protein folding predicted by theory, and are now offering the first glimpses at the underlying mechanisms. Fast folding appears to also have functional implications as recent results connect downhill folding with intrinsically disordered proteins, their complex binding modes and ability to moonlight. These connections suggest that the coupling between downhill (un)folding and binding enables such protein domains to operate analogically as conformational rheostats.

## BACKGROUND

**Figure 1:**
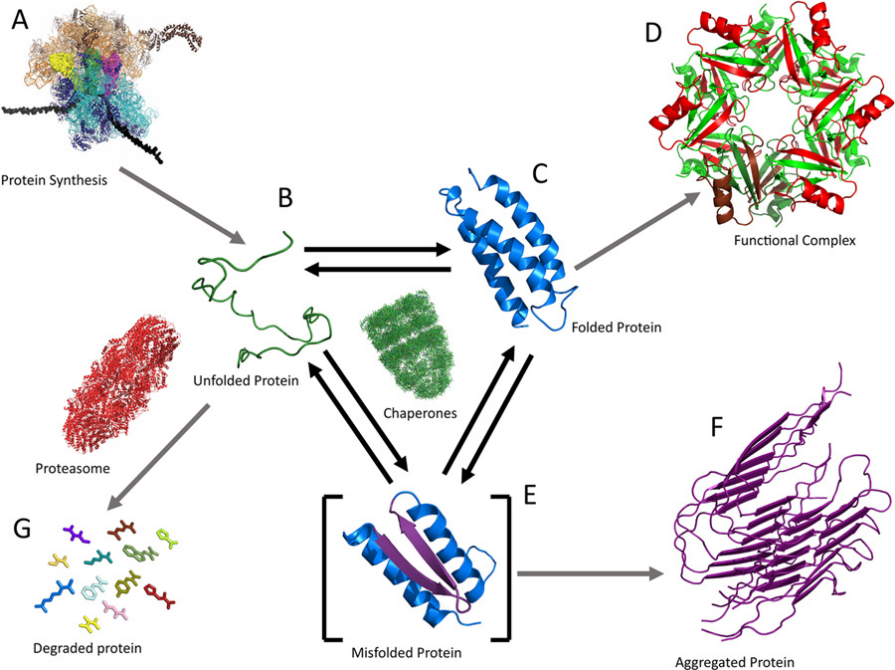
Protein folding inside the cell A new protein is synthetized at the ribosome as determined by the activation of gene expression. The nascent chain is typically bound to chaperones that keep it unfolded until the process is complete and the chain is released (**A**). The recently synthesized unfolded protein folds autonomously and reversibly, establishing a dynamic equilibrium between the unfolded ensemble (**B**) and the native state (**C**). This conformational equilibrium determines the protein's ability to perform its function, either directly as a monomer or by further assembling into larger macromolecular complexes (**D**). The half-life of its biological activity is also controlled by the folded–unfolded equilibrium because the proteasome machinery eliminates targeted proteins from their unfolded state (**G**). Likewise, the transient formation of partially folded conformations can lead to misfolding events (**E**) that feed the formation of aberrant and/or toxic protein aggregates (e.g. amyloids) (**F**). The ribosome image is reproduced with permission from Schmeing and Ramakrishnan (2009) Nature **461** 1234–1242. All other structures are available from the PDB.

Proteins are true nanomachines in charge of most biological roles in living cells, a feat they accomplish by self-assembling into sophisticated 3D structures that exploit thermal, and on occasion chemical, energy to change shape in response to stimuli. As proposed by Anfinsen [1] in his seminal work, the process by which proteins fold into their functional structures is dictated by the chemical blueprints encoded into their amino acid sequence. This assertion implies that if we understood protein folding mechanisms in depth, we would have at our fingertips the ability to read genomic information in real functional terms, and to design and engineer synthetic biological components *à la carte*. Such motivations have fuelled the interest in the ‘protein folding problem’ among scientists from a wide variety of disciplines. Moreover, the physical principles that govern protein self-assembly still hold true *in vivo*, where folding is tightly coupled to many other processes that control the protein life cycle (Figure 1). In fact, the protein cycle shown in Figure 1 emphasizes that understanding the mechanisms of protein folding is an essential requirement to comprehend protein homoeostasis *in vivo*. It is thus our contention that molecular biologists and biochemists ought to keep abreast of recent exciting developments in protein folding research, even if those developments are increasingly coming from the realm of physics.

In this regard, the development of ultrafast folding approaches was a major turning point in protein folding research. Previously, experimentalists were constrained by the millisecond resolution of stopped-flow methods, which also offered limited structural resolution. The single-domain proteins that could be studied folded slowly (from tens of milliseconds to minutes) and apparently via a single stroke process, which led to the generalization of the two-state folding model [2]. Although apparently simple two-state folding implies that all of the intermediate structures responsible for defining the mechanism are highly unstable and thus inaccessible to experiment. In a parallel front, analytical theory based on condensed matter and polymer physics defined folding reactions as the stochastic search for the native structure on a corrugated hyper-dimensional energy landscape with an overall funnelled shape that acts as driving force (the energy landscape approach) [3]. Such description had three key implications that departed drastically from conventional interpretations of folding: (i) the existence of myriad microscopic folding pathways instead of a unique sequence of structural events [4]; (ii) the definition of a folding speed limit determined by the timescale of the relevant conformational motions of the protein [5]; and (iii) the prediction that folding free energy barriers originate from entropic bottlenecks and are in general shallow, leading to the possibility of downhill (barrierless) folding [6]. At the time, theory could only be tested against computer simulations that used coarse-grained representations of protein structure [7–9] since the best atomistic simulations were still six or seven orders of magnitude too short in their accessible timescales [10]. Coarse-grained simulations supported energy landscape predictions and showed that a two-state mechanism was not required for efficient folding [11]. There was, however, a deep divide between what theory and simulations predicted and what experiments reported.

Access to novel ultrafast folding methods drastically changed this state of affairs, permitting to connect experiments, theory and simulations in synergistic ways. In the present review, we discuss the key contributions as well as the methodological developments that made them possible. In so doing, our goal has not been to be fully comprehensive. After all, the material is too extensive and there already are excellent technical reviews that cover many of these topics separately. Our major motivation has been instead to provide the non-expert reader with an account of the most compelling discoveries in this area and of their significance in shaping the solid conceptual framework in protein folding that we enjoy today. We end the review discussing recent exciting results that highlight the functional and technological implications of fast protein folding as molecular mechanism for the implementation of conformational rheostats.

## METHODS TO INVESTIGATE ULTRAFAST FOLDING

Fast folding is defined as any protein conformational change that takes place in less than 1 ms. In this section, we describe different approaches that reach the sub-millisecond timescales required to investigate fast-folding reactions.

### Ultrafast kinetic techniques

**Figure 2:**
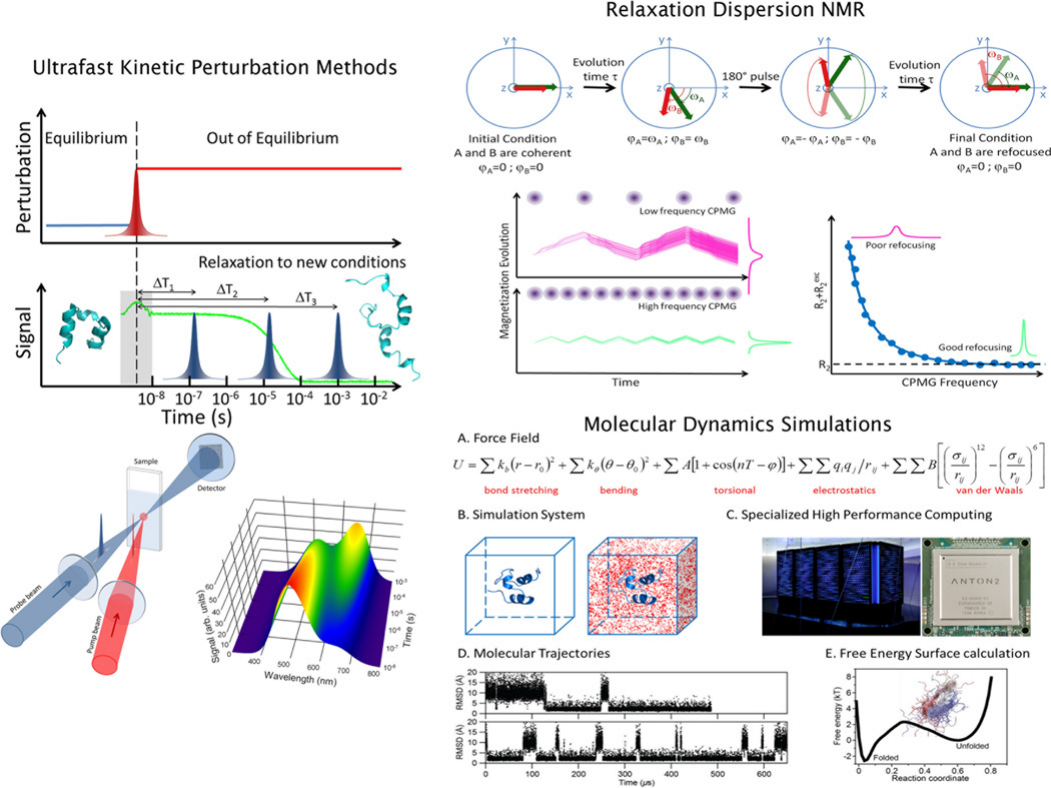
Experimental and computational approaches for investigating fast protein folding Ultrafast kinetic perturbation methods (left): these techniques report on the macroscopic (bulk) relaxation decay to the new thermodynamic conditions imposed by fast perturbation. The key is to devise an efficient procedure to quickly alter the folding–unfolding equilibrium by changing temperature, pH, pressure or chemical potential. The relaxation is monitored using spectroscopic techniques that lead to the determination of the relaxation rate and the amplitude of the change in signal, which, when analysed with a suitable kinetic model, provide the changes in population and the microscopic rates of interconversion between species. The fastest of these methods use laser pulses as triggers, resulting in time responses potentially as short as 1 ps (10^−12^ s). The laser-induced T-jump method represented produces 10–15°C jumps in less than 10 ns. Relaxation dispersion NMR (top right): different nuclei in the protein rotate (precess) about the magnetization axis at slightly different frequencies given by their chemical shifts, resulting in a loss of phase coherence that broadens the overall magnetization signal that is recorded on the transverse plane (*x*–*y* plane). In a system in which chemical shifts do not change with time, the signal decays according to the transverse relaxation rate (R2), which for proteins is ∼5–20 s^−1^. The interconversion between species in shorter times results in further decoherence, and thus in broader signals/faster decays (enhanced relaxation). The RD-NMR experiment measures this effect using specific radiofrequency pulses that flip the magnetization 180° on the *x*–*y* plane to induce refocusing because the faster spins, which are now behind, will eventually catch up with the slower ones. The refocusing pulses cannot compensate for changes in chemical shift due to the molecules exchanging conformation during acquisition. Therefore applying trains of refocusing pulses interspersed at fixed times (Carr–Purcell–Meiboom–Gill or CPMG relaxation dispersion [33]) makes it possible to measure conformational exchange processes. Molecular dynamics simulations (bottom right): MD trajectories of proteins and their analysis are performed in five steps: (A) defining a molecular mechanics force field that calculates the potential energy of the protein as a function of the atomic co-ordinates; (B) building a simulation box that contains all of the atoms from the protein (atomic co-ordinates) plus surrounding solvent (water) molecules; (C) numerical integration of Newton's equations of motion defined by the positions, forces and velocities of every atom in the simulation box over time steps of ∼1 fs (10^−15^ s) using a supercomputer (shown here is Anton, the computer designed by D.E. Shaw Research for ultra-efficient MD simulations); (D and E) analysis and molecular-mechanistic interpretation of the terabytes of data included in the simulated trajectories.

Kinetic experiments measure the conformational relaxation of the protein in response to a perturbation, and have time resolution determined by how quickly the perturbation is enacted (Figure 2). The first fast-folding experiment used a laser trigger to rapidly initiate folding of chemically denatured cytochrome *c* by photodissociating the haem-bound carbon monoxide, which binds preferentially to the unfolded state [12]. Several photochemical triggers have been used for fast-folding research [13–15], but they are usually protein-specific. An alternative is to use laser pulses to change the surrounding solvent. Photochemical stimulation of caged compounds added to the solution, such as *o*-nitrobenzaldehyde, releases one proton per molecule resulting in abrupt pH decreases (up to ∼2 units) in ∼100 ns [16]. Ultrafast temperature jumps can be induced by heating the surrounding solvent with an infrared laser pulse at a frequency that overlaps with water vibrational modes [17–19]. The laser-induced T-jump technique attains increases of ∼10–15°C in a few nanoseconds (100000-fold faster than stopped flow), and is universal because protein folding reactions are always temperature-dependent [20]. These characteristics have made the laser-induced T-jump technique the most popular ultrafast kinetic method, including implementations that detect fluorescence intensity [17,19], fluorescence spectra [21], infrared absorption [22,23] and Raman scattering [24]. More recently, this technique has been combined with selective isotope editing to monitor fast-folding kinetics with nanosecond resolution at the level of single peptide bonds [25] and individual side chains [26].

Another ultrafast kinetic perturbation method induces pressure jumps that unfold proteins due to the larger total volume occupied by the native protein and its hydration shell [27]. The fastest implementation of this method achieves microsecond resolution using an electrical discharge to rupture a metallic membrane separating the protein sample from a solution held at very high pressure [28,29]. Advances in chemical dilution techniques using rapid mixers in continuous flow operation have also led to resolutions of ∼50 μs for turbulent mixing [30,31] or only 10 μs for set-ups using microfluidics and hydrodynamic focusing [32].

### Single-molecule spectroscopy

Single-molecule methods can resolve the stochastic conformational fluctuations of the protein in equilibrium conditions, and thus do not need a fast perturbation. Time resolution is simply determined by the speed of data acquisition. Single-molecule methods available to investigate protein folding can be classified into two groups: (i) force spectroscopy techniques in which single protein molecules are unfolded by pulling from its ends and refolded upon releasing the force; (ii) enhanced optical microscopy methods that detect individual proteins while they undergo conformational transitions, typically using fluorescence imaging (single-molecule fluorescence microscopy).

Mechanical unfolding can be achieved with a variety of methods, such as atomic force microscopy [34], magnetic tweezers [35] and optical tweezers [36,37]. Each of these techniques has advantages and disadvantages [38]. Data collection is limited by the oscillation frequency and spring constant of the microdevice that exerts mechanical control (e.g. AFM cantilever or the microbeads in an optical tweezers set-up), which constrains most of these techniques to millisecond resolutions. There are, however, a few applications of optical tweezers that are now reaching microsecond resolution [39,40].

Single-molecule fluorescence spectroscopy employs a confocal microscope to illuminate volumes of only ∼1 fl (10^−15^ litres) to isolate and detect individual protein molecules, whether immobilized on a surface or freely diffusing. The microscope objective also collects fluorescence photons emitted by the molecule, which are detected by highly efficient, picosecond resolution, avalanche photodiodes [41]. Information about conformational transitions is commonly obtained from the efficiency of FRET (Förster resonance energy transfer) between a donor–acceptor fluorescent pair introduced in specific positions of the protein [41,42]. Time resolution is not set by the detector's response, but by photon emission statistics; that is, by the time it takes to collect the bunches of approximately 50 photons required to measure FRET efficiency (i.e. the ratio between number of photons emitted by the acceptor and total number of emitted photons) with reasonable accuracy [43]. The characteristically low photon collection efficiency of these methods (∼1% [44]) and the low illumination intensity required to avoid photochemical damage of the fluorescent dyes has typically limited their resolution to milliseconds [45]. However, implementation of ultra-efficient photoprotection cocktails have raised the resolution up to ∼50 μs [46]. A complementary approach involves developing methods to analyse photons one by one, like the Gopich–Szabo MLA (maximum likelihood analysis) of photon trajectories [47,48]. Recently, the combination of this approach and a simple theoretical model of protein folding has shown promise to extend the time resolution to 10 μs [49].

### Probing fast folding at atomic resolution

NMR (nuclear magnetic resonance) is particularly attractive for protein folding studies because it provides both atomic-resolution and dynamic information [50]. Transverse RD-NMR (relaxation dispersion NMR) experiments (Figure 2), in which the NMR signal decoherence caused by chemical shift anisotropy is measured as a function of time, are particularly advantageous. RD-NMR can detect exchange with partially folded conformations that are only minimally populated (down to 0.2%, the so-called invisible states) from the broadening of the native state peaks [51]. Moreover, it permits the extraction of the exchange rate, the population of the invisible species and also its chemical shift values [52]. Chemical shifts provide structural information about the invisible state at atomic resolution [53]. In this technique, time resolution is ultimately determined by technical limitations (e.g. how quickly the refocusing pulses can be applied) that make it ideal for millisecond processes [54]. However, recent developments have pushed the RD-NMR resolution down to 200 μs (rates of 5000 s^−1^) [55].

### Atomistic computer simulations

MD (molecular dynamics) simulations offer atomistic structural resolution and dynamic information with virtually infinite time resolution. Therefore MD simulations could potentially provide all of the structural and dynamical insights required to understand the underpinnings of folding mechanisms [56] (Figure 2). Here the problem is not time resolution, but how to reach the much longer timescales of protein folding reactions. For decades, the enormous timescale gap that existed between MD simulations and folding experiments impeded testing and refining the simulations with empirical data and also restricted their use in interpreting experimental results. The first all-atom MD simulation in explicit solvent to hit the 1 μs mark was performed by Duan and Kollman [57] on the small villin headpiece subdomain. The simulation did not achieve complete folding, but it captured hydrophobic collapse and helix formation in the unfolded state [57]. In the last decade, major technical advances have increased sampling to the level of resolving multiple folding–unfolding events on several fast-folding proteins (see [58] for a detailed review). Such approaches have been of two kinds. In the first one, sampling is enhanced by running thousands of short (∼1–5 ns) MD simulations using distributed computing strategies [59]. The idea is to capture some of the fastest possible folding trajectories of a fast-folding protein by taking advantage of the exponential distribution of folding times [for a process with folding rate of 1/(1 μs), the probability of seeing a 1 ns folding trajectory is ∼0.1%]. The second approach aims at making the calculations faster, by optimizing the code [60], by optimizing the force field [61] or by designing new computers hardwired for MD calculations [62]. The latter have resulted in the implementation of MD simulations that routinely reach the 1 ms mark [63], which in turn permits the optimization of force fields by thorough testing and comparison with folding experimental data [64].

## MAPPING PROTEIN FOLDING MOTIONS

**Figure 3:**
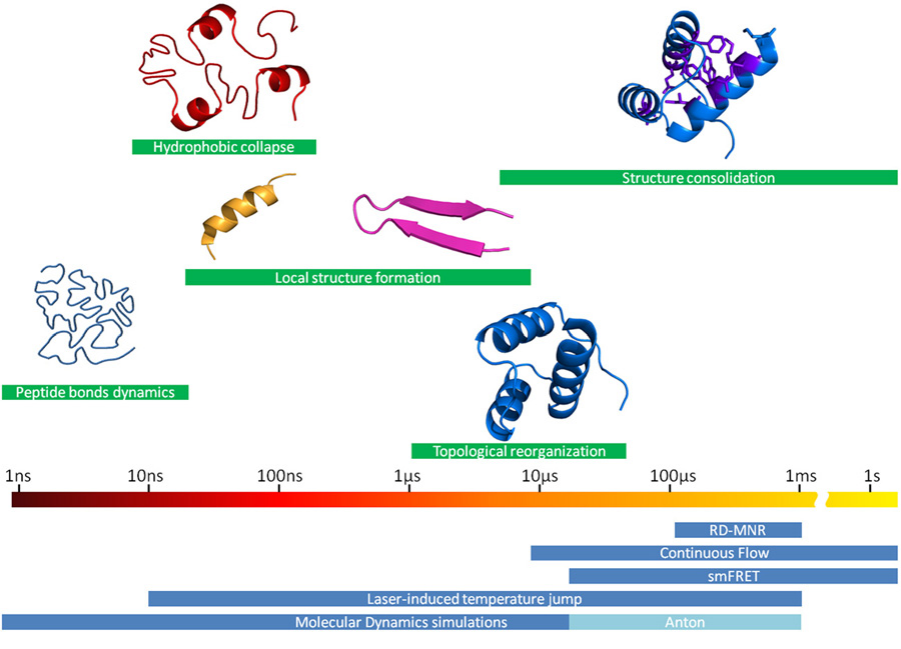
Roadmap of folding timescales The chart shows the timescales associated with the various structural events that take place in protein folding reactions and the experimental and computational methods that are used to probe them. smFRET, single-molecule FRET.

A major contribution from ultrafast kinetic methods has been the determination of the timescales for various folding-related conformational motions [65]. Combining seminal contributions from multiple groups, we can now establish a basic roadmap of the timescales that are relevant to the different structural events that take place during folding reactions (Figure 3).

### Hydrophobic collapse

The random collapse of the unfolded polypeptide chain to exclude hydrophobic side chains from surrounding solvent is one of the key events that take place during folding. Collapse was theoretically predicted to be much faster than folding [66], which immediately made it a target for fast-folding experiments. Seminal laser T-jump experiments on a pH-denatured small protein that collapsed as a function of temperature rendered timescales of 100 ns [21]. This nanosecond timescale was later confirmed on other denatured proteins using single-molecule spectroscopy [67]. Therefore non-specific hydrophobic collapse appeared to be among the fastest folding-related processes [21]. The rate, however, can be decreased significantly when collapse is initiated from the fully expanded unfolded state by chemical denaturant dilution [68]. Later efforts have focused on determining the role that interactions other than the hydrophobic force exert on the properties of unfolded polypeptides. Experiments on IDPs (intrinsically disordered proteins) are noteworthy because these proteins do not fold, have low hydrophobicity and have high charge density [69]. Interestingly, single-molecule FRET experiments performed on IDPs also showed a more compact state in the absence of chemical denaturants, indicating that backbone hydrogen bonds also play a role in the solvent-induced contraction of unfolded polypeptide chains [70,71].

### Loop formation

The closure of loops determines the formation of interactions between secondary-structure elements to form supersecondary and tertiary arrangements. The timescale for loop formation was accordingly considered a proxy for the folding speed limit [72], a parameter that is important for determining the magnitude of folding barriers from the experimental rates (see the next section). The inaugural fast-folding experiment involved the determination of the rate of closing a long loop in chemically unfolded cytochrome *c*, which occurred in tens of microseconds [12]. Using scaling arguments, these results led to an estimate of 1 μs for the formation of shorter protein-like loops [72]. Subsequent experiments on short flexible peptides in aqueous solution have produced timescales for forming protein-like loops that are much shorter, ranging from ∼50 ns for contact formation between the ends of 12–20-residue unstructured peptides [73,74] down to only 10 ns for contact formation on the shortest three- or four-residue turns [74].

### Secondary structure

Investigating the timescales for secondary-structure elements required combining the fastest kinetic methods with small peptides that were able to form stable secondary structures on their own. α-Helix formation was studied early on using alanine-rich peptides and found to occur in ∼150 ns [18,19]. Subsequent studies used photoswitches to trigger helix formation from chemically distorted non-helical conformations [14,75]. Interestingly, for richer amino acid sequences (more protein-like) helix formation was found to be only slightly slower (a factor of 2–3) [76]. Although the α-helix is a simple and symmetric structure, higher-resolution experiments showed that helix formation occurs via a complex kinetic process in which nucleation, propagation and the splitting and merging of helical segments conspire to produce different timescales for different positions along the molecule [77–80]. Nevertheless, such complexity takes place within a narrow range of timescales: 150–500 ns for the formation of stable α-helices, and up to ∼1.2 μs for the nucleation of a thermodynamically unstable helical turn on cyclized peptides [81]. The similar timescales for non-specific collapse and α-helix formation suggest that both processes occur almost concomitantly during the early folding stages.

The β-hairpin is the minimal β-structure and the basic component of antiparallel β-sheets. Moreover, β-hairpin formation includes local structure (the turn) and collapse of the strands, and thus can be considered the simplest example of protein folding (i.e. ‘mini-protein’). The first experiments on β-hairpin formation were performed on the GB1 hairpin, which was naturally stable when isolated from the protein [82,83]. These experiments showed a relaxation time that was almost 25-fold longer than α-helix formation. Statistical mechanical modelling could neatly explain the differences between both types of structures by invoking a mechanism in which β-hairpins form the central turn first and then zip up the two strands [82,83]. These seminal results and conclusions elicited a great deal of interest in the investigation of β-hairpin formation, which became a benchmark for protein folding studies. A vast array of computer simulations on β-hairpin folding ensued [84–93], producing results that tended to favour the opposite mechanism in which the hydrophobic cluster collapsed first followed by zipping down to form the turn last. However, subsequent experiments confirmed the main conclusions from the original GB1 β-hairpin study. For example, it was shown experimentally that β-hairpin folding is accelerated when the loop connecting both strands is shortened [94], and that turn formation plays a key role in driving β-hairpin folding [95,96]. More recently, an exploration of the speed limit for β-hairpin folding has demonstrated that, when the turn is autonomously stable, β-hairpin formation takes only ∼100 ns, approaching the timescales for the fastest helix formation [97].

### Topological reorganization

The rearrangement of secondary-structure elements to form native tertiary interactions on a randomly collapsed globule should occur intrinsically more slowly than the motions described before because it requires breaking pre-formed interactions before the protein can reconfigure. Frieden and co-workers measured the reconfiguration dynamics of an unfolded protein under different solvent conditions and found that such conformational rearrangements take place in a few microseconds, and involve formation and dissolution of partially folded structures [98]. Additional experiments by Lapidus et al. [99] with newer and faster continuous flow mixers (20 μs mixing time) have shown that, whereas non-specific collapse occurs within the instrument dead-time, formation of native tertiary contacts is the last event requiring times of at least 100 μs.

## FAST PROTEIN FOLDING

### Understanding the determinants of protein folding rates

Figure 3 provides an entry point to investigate the determinants of the over six orders of magnitude spread in folding rates that is observed in natural single-domain proteins [100], and, as an ancillary issue, to estimate the speed limit for protein folding reactions [101]. For these purposes, we can utilize a simple folding rate expression derived from the energy landscape approach [6]:

1 $$k_{\mathrm{fold}}=\frac{1}{\tau_{0}}\exp\left(-\Delta G^{†}/RT\right)$$

where Δ*G*^†^ is the overall free energy barrier to folding and τ_0_ is the timescale for the conformational motions undergone by the protein when is crossing the barrier top. The pre-exponential term should be relatively protein invariant, and thus the large differences in folding rates must come from the free energy barrier. According to theory, the barrier arises from an abrupt loss in conformational entropy that occurs at early stages and is not compensated by formation of stabilizing interactions until much later in the process [4]. On the other hand, the folding speed limit is determined by the pre-exponential term and is achieved when the free energy barrier vanishes, resulting in downhill (barrierless) folding [6,102].

**Figure 4:**
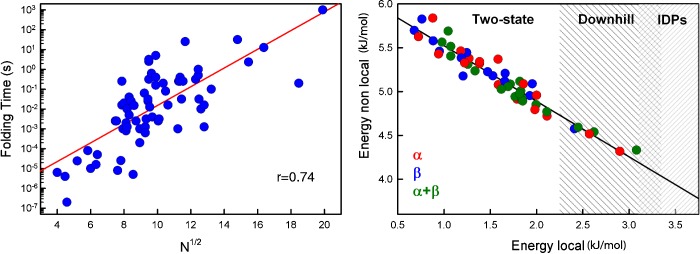
The determinants of protein folding rates Left: the direct correlation existing between protein folding times and a fractional exponent of the number of amino acids in the protein (in this case ½). Right: the compensation between the stabilization energy per residue provided by local and non-local interactions. The higher the non-local fraction the lower the folding rate.

Figure 3 suggests that the folding speed limit lies between 10^7^ and 10^5^ s^−1^ (τ_0_ from 100 ns to 10 μs). Using this range, it was possible to estimate barrier heights from experimental folding rates, and derive its entropic and enthalpic contributions [103]. This empirical analysis confirmed that folding barriers are entropic bottlenecks as predicted by theory. Moreover, folding rates scale with the size of the protein, which results in inverse correlations between experimental folding rates and a fractional exponent of the number of amino acids [100,104,105] (Figure 4, left). Interestingly, the slope of the rate against size correlation rendered an independent estimate of the folding speed limit of 1 μs [100], which is in the middle of the range estimated from Figure 3, and identical with the estimate obtained from loop formation in cytochrome *c* (see above).

Folding rates also depend on the properties of the native 3D structure. This factor was first observed in a correlation between experimental folding rates and the contact order, a parameter related to the average sequence separation between residues in close native contact [106]. The relationship between rates and native structure was later rationalized theoretically using statistical mechanical modelling of protein folding [107]. Size also plays an important role in determining protein stability [108] and unfolding rates [109]. In fact, just ten bits of protein-specific information (number of amino acids and structural class) seem to be sufficient to predict folding and unfolding rates (and thus stability) with errors of magnitude equivalent to the typical perturbations induced by just two point mutations [109]. The combined effects of size and structural class can be condensed on to a single parameter that defines the fractional contributions to native stability from local (between residues close in sequence) and non-local (tertiary) interactions (Figure 4, right). The local fraction increases as the protein becomes smaller because the higher surface/volume ratio of smaller globular structures results in fewer tertiary contacts per residue.

### Searching for microsecond folding proteins

New kinetic techniques also opened the opportunity to resolve the folding–unfolding kinetics of proteins that were too fast for the stopped-flow method. This possibility triggered a quest for the fastest possible protein with the goal of reaching the folding speed limit [101,110], and thus the downhill folding scenario [111]. Inspired by the concepts exemplified in Figure 4, efforts focused on either small protein domains or proteins with maximal energetic contributions from local structure. As a consequence, a large number of proteins that fold to completion in just microseconds has been identified over the last few years [65,112] (see the left-hand panel of Figure 4). Most of these proteins are α-helix bundles [110,113–118] or very small antiparallel β-proteins, such as WW domains [119–122], although there also are examples of mid-size domains with mixed α/β structure [123]. Additional efforts have focused on trying to increase the folding rate by increasing the net stabilization from local interactions, including strategies such as weakening the hydrophobic core of λ-repressor [110], engineering WW domains with very stable β-turns [124,125] or looking at larger *de novo* designed proteins with maximal helical propensity such as α_3_D [115].

### Downhill folding

Another exciting implication of fast folding research is the downhill folding scenario. Barrierless folding raised a lot of interest because it was an opportunity to test experimentally a true prediction from energy landscape theory that could not be explained with traditional descriptions [102,126]. Moreover, on a downhill folding protein, the partially structured intermediates on the folding pathway could be populated and thus become accessible to experimental detection [111]. The problem was how to unambiguously diagnose downhill folding without relying exclusively on timescales. Early attempts focused on the observation of strange kinetics (relaxations over-extended in time) that could be due to the transient accumulation of large numbers of intermediates. Strange kinetics was indeed reported from T-jump experiments that induced refolding from the cold-denatured state of several slow-folding proteins, such as yeast phosphoglycerate kinase and a mutant ubiquitin [127], or a cold-shock protein from *Escherichia coli* [128].

Later, it was appreciated that thermodynamics provided much more reliable diagnostics than kinetics because a downhill folding protein should (un)fold gradually resulting in structurally complex equilibrium unfolding [129]. The combination of multiple spectroscopic probes, calorimetry and theoretical analysis revealed such gradual unfolding on the small helical protein BBL, which was accordingly identified as the first example of global downhill folding [130]. This classification was subsequently confirmed by measuring the thermal unfolding of BBL at atomic resolution using NMR [131]. Subsequent kinetic studies using multi-probe laser T-jump experiments demonstrated that BBL folds extremely fast (folding time of ∼1 μs at the midpoint temperature), as expected [117]. The connection between how broad and heterogeneous is the equilibrium unfolding of a protein and how fast it folds was elegantly demonstrated in follow up experiments that compared BBL with the ∼10-fold slower structural homologue PDD [132]. A more recent test that relies on measuring folding kinetics after T-jumps of different magnitude to the same final temperature has confirmed the one-state downhill folding of BBL in contrast with the also fast, but barrier-crossing, folding of Trpzip-3c [133].

Other groups have pursued the downhill folding regime by increasing the rate of fast-folding proteins via mutation. Gruebele and co-workers engineered the microsecond folder λ_6–85_ to make it even faster, and thus induce the emergence of the probe-dependent equilibrium unfolding and complex kinetics of the global downhill folding regime [134–136]. Eaton and co-workers took the villin headpiece subdomain, a small helical protein that folded in few microseconds, and sped up folding 6-fold with two designed point mutations [137]. This superfast villin version was the first example of sub-microsecond folding, and was subsequently identified as a downhill folder from multi-probe and kinetic criteria [138].

The most direct observation of the gradual disordering associated with global downhill folding has been obtained with modern single-molecule fluorescence spectroscopy [139]. In principle, single-molecule methods can directly distinguish between a scenario in which each molecule is either fully unfolded or folded (barrier-crossing folding) and a scenario in which individual molecules are partially unfolded (global downhill folding) [46,139,140]. The challenge was how to make the photon count rates much higher than the ultrafast relaxation expected for a downhill folder. In this case, the authors managed to do so combining advanced photoprotection methods [46] and measurements at very low temperature to slow down the BBL folding–unfolding relaxation 100-fold [139].

## PROBING ENERGY LANDSCAPES OF PROTEIN FOLDING

The multidimensional energy landscapes of protein folding are supposed to be funnelled towards the native 3D structure, but also rugged (many local valleys and peaks) [102,141]. Landscape topography is in fact very important because it determines the specific folding pathways and mechanisms for each protein, but it has proved to be extremely difficult to probe experimentally. For slow two-state folding domains, the only method available is mutational analysis in which the effects that mutations have on both folding and unfolding rates are used to infer structural properties of the folding transition state ensemble (the conformations that make the barrier top in eqn 1) [142]. This method has been widely used, and there has been ample discussion about the mechanistic interpretation of such mutational data [143–146]. More recently, large-scale analysis of the wealth of mutational data available on multiple proteins has revealed an almost universal trend (one-third of the free energy change on folding and two-thirds on unfolding) [147], which indicates that the specific structural and mechanistic information included in these datasets is very limited [147]. On the other hand, the discovery of fast-folding proteins has made it possible to develop powerful alternatives based on a new set of methods for probing the topographic details of folding energy landscapes.

### Thermodynamic folding barriers from calorimetry

DSC (differential scanning calorimetry) is extremely sensitive to the conformational heterogeneity of protein folding reactions [148]. DSC can thus be employed to estimate the shape of the folding free energy surface. Such a method was originally developed to distinguish between all-or-none (two-state) and gradual (downhill) protein folding [149]. It was subsequently demonstrated that the DSC experiment has enough information to detect small deviations from two-state caused by minimal population (<1%) of the barrier top conformations, leading to the possibility of estimating folding free energy barriers from these thermodynamic experiments [150]. DSC has thus become a powerful tool for characterizing folding energy landscapes, especially for proteins that fold fast [123,132,151,152] or with limited co-operativity [153,154]. The method has solid theoretical grounds, but its practical implementation requires fitting the DSC data to a specific model that represents the folding free energy surface [148]. The results are somewhat dependent on how well the model can represent the underlying conformational ensemble. A recent workaround involves analysing the DSC data with various theoretical models that are then ranked with a Bayesian probabilistic approach to obtain model independent estimates of the folding barrier height [155].

### Reconstructing folding landscapes from multi-probe unfolding experiments

Fast folding proteins exhibit marginal unfolding co-operativity that results in non-concerted structural disassembly [156]. The decoupling between structural elements can be resolved using multiple spectroscopic probes [129]. The folding landscape is then reconstructed interpreting all the spectroscopic data with statistical mechanical models that include the most relevant partially folded conformations. This approach, which was originally developed to identify one-state downhill folding [129,130], is, in principle, extensible to any fast-folding protein. Such extensibility has been demonstrated by various groups that applied it to reconstruct the folding landscape of villin headpiece subdomain [157], PDD [158], the *de novo* designed αtα [159], and the P22 subdomain [160]. In recent applications, the multi-probe data is combined with kinetic information [157] or enhanced by incorporating residue-specific infrared probes [159,160]. All of these studies have rendered free energy landscapes that are broad and with shallow barriers, but have also revealed differences that highlight distinctive mechanistic features. For example, comparative analysis between structural homologues (BBL compared with PDD [158] and αtα compared with P22 [160]) showed biases in the relative stability of different structural elements on proteins that share the overall fold.

### Folding interaction networks at atomic resolution

The non-concerted unfolding behaviour of fast-folding proteins can be taken one step further to determine the network of interactions that stabilize the native structure [161]. Here NMR is used to measure the chemical shifts of every relevant atom in the protein at different degrees of unfolding, induced, for example, by increasing temperature [131,161]. The experiments produce hundreds of different chemical shift curves that report on the local changes in electronic environment of the corresponding atoms during unfolding. Such atomic unfolding curves are highly heterogeneous, but collectively represent the global unfolding process [131]. Because the similarities between unfolding curves from pairs of residues is related to their degree of structural coupling during unfolding, such information can be used to infer the folding interaction network [161]. This method was first implemented on the downhill folder BBL, which showed an extremely broad distribution of atomic unfolding behaviours [131]. Recently, it has been successfully extended to the fast folder gpW, demonstrating that is not exclusive of global downhill folding [162]. The gpW data were less heterogeneous and revealed a structurally delineated network of couplings between residues scattered throughout the sequence [162]. For gpW, long-timescale MD simulations at various temperatures were performed in parallel to reproduce the entire unfolding process. From the atomistic trajectories, it was possible to compute chemical shift unfolding curves and thus derive a simulated folding interaction network to be compared directly with the experimental one [162]. The possibility to compare experiments and simulations at this high level of detail is key for the interpretation of experimental data in mechanistic terms and for benchmarking and refining simulation methods.

### Structural analysis of excited states in protein folding

RD-NMR (see above) has been widely used to detect minimally populated species associated with protein conformational changes taking place during catalysis, ligand binding or DNA sliding motions, which tend to occur in the sub-millisecond to millisecond timescale [54]. Likewise, NMR has been increasingly used to resolve the structure of folding intermediates on slow three-state folding proteins [51,163]. Some of these intermediates, like for the 71-residue four-helix bundle FF domain [164], form relatively fast (sub-microsecond), indicating a process that crosses a very small barrier. Chemical shift analysis indicated that such fast-forming intermediates have a compact structure with non-native interactions that need to break before the native state appears in a much slower step [165], thus being examples of kinetic traps [6].

When applied to fast-folding proteins, RD-NMR methods could provide detailed structural information of the conformations corresponding to the top of the folding barrier. In contrast with intermediates that accumulate before the rate-limiting step, the barrier top species determine the overall folding rate and hold the keys to the mechanism. The major challenge is the timescale of the process, which needs to be longer than 0.1 ms to be resolvable with these methods. Recently, a full-blown RD-NMR characterization of the ultrafast folder gpW has been achieved at very low temperature (1°C) to decrease folding kinetics down to ∼4000 s^−1^ [55]. Under these native conditions, the most populated non-native conformations are expected to be those that sit at the top of the shallow barrier that is found at the denaturation midpoint [55]. RD-NMR experiments revealed an exchange process with rate identical with the overall folding rate in which the excited state had a population of ∼10% and a structure with the two native helices in gpW formed and the β-hairpin unfolded [55]. These RD-NMR experiments are arguably the first example of high-resolution structural analysis of the conformations that determine the folding rate and mechanism of a protein. Interestingly, the structural properties of the barrier top derived by RD-NMR were in very close agreement with the experimental analysis of the folding interaction network and the long-timescale MD simulations obtained independently on the same protein [162] (see the previous section).

### Folding pathways and mechanisms

**Figure 5:**
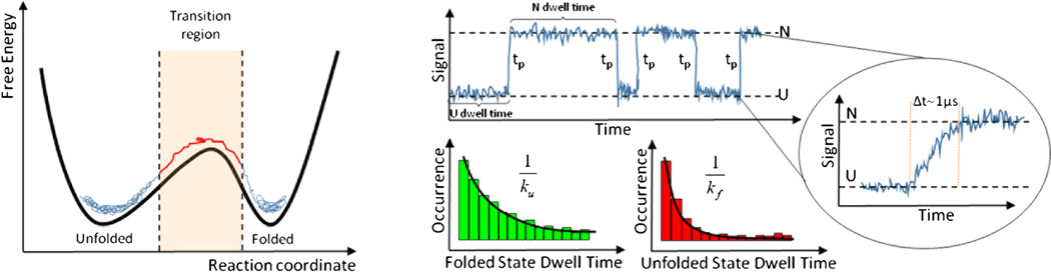
Transition paths in protein folding The folding free energy landscape of a single-domain protein is often represented with a simple 1D surface with two minima for the native (N) and unfolded states (U), and a more or less pronounced barrier separating them (left). On this surface, individual molecules dwell on either minimum for most of the time since climbing the barrier is a probabilistically rare event. However, when it happens, the transitions across the barrier are fast because they are only limited by the conformational motions at the barrier top. This results in single-molecule trajectories that slowly alternate between U and N with very sharp transitions (right). The average dwell times on U and N are equivalent to the inverse of the folding and unfolding rate constants measured in bulk kinetic experiments respectively, whereas the sharp transitions correspond to the barrier-crossing paths.

In addition to obtaining structural information, it is also important to measure dynamic events such as folding transitions and microscopic pathways because they reveal the heterogeneity of mechanisms. For a barrier-crossing process, folding transition paths are the conformational excursions that take the protein over the barrier (Figure 5). Transitions occur very rarely, but are extremely fast. In fact, the typical time the molecule spends crossing the barrier is related to the pre-exponential factor of eqn 1. Therefore one would expect folding transition paths to take a few microseconds and be broadly distributed. Resolving individual transition paths requires methods that simultaneously reach single-molecule, sub-microsecond and atomic resolutions. Not only that, but also the observation times need to be sufficiently long to catch these rare events (Figure 5).

The first pass at estimating folding transition path times experimentally came once again from fast-folding proteins [110]. As implemented by Gruebele and co-workers, the idea was to look for evidence of an even ‘faster’ minor kinetic phase in T-jump experiments of proteins that were already near the downhill limit [110,112]. Such a process, termed the molecular phase, should correspond to the depopulation of the barrier top in response to the perturbation. The molecular phase has been observed in several fast-folding proteins and their mutants, including the helix bundle λ_6–85_ [110,166] and the WW domain FiP35 [167]. The timescale for this process (measured at relatively high temperatures of ∼60–70°C) is 1–2 μs, in line with other estimates of the folding speed limit.

Eaton and co-workers have attempted to measure transition paths directly with single-molecule fluorescence methods to obtain estimates of the average folding transition path time for several proteins [168,169]. The time-resolution limitation was overcome by slowing down folding dynamics by addition of viscogens and analysing the single-molecule trajectories photon by photon [47]. The average transition path time of two natural proteins, one that folds fast and another one that folds slowly, was found to be very similar (between 2 and 10 μs) and consistent with previous estimates of the folding speed limit [168]. In contrast, the folding transition path time of the *de novo* designed α_3_D protein was much longer [169] even though this protein folds very fast [170] and close to the downhill limit [171]. Further analysis and comparison with MD simulations has revealed that the barrier crossing for this protein involves formation of off-register hydrogen bonds between the helices that need to break to proceed towards the native state, which increases internal friction and thus slows down the pre-exponential term [172].

**Figure 6:**
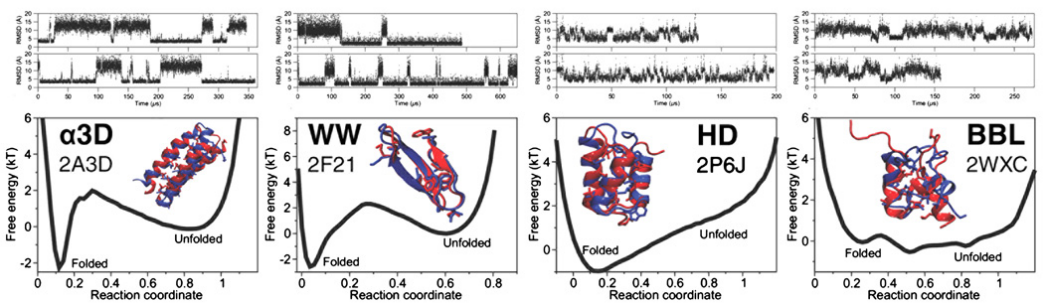
Long-timescale MD simulations of fast-folding proteins MD simulations on the proteins α_3_D (2A3D), WW domain (2F21), engrailed homedomain (HD, 2P6J) and BBL (2WXC). The upper panels show two individual MD trajectories for each protein revealing multiple folding and unfolding events. The lower panels show the 1D free energy surfaces derived from the MD simulations together with the superposition of the experimentally determined native structure (red) and the native structure identified by the simulations (blue). The free energy surfaces highlight that some fast-folding proteins cross very small free energy barriers (α_3_D and WW) and others fold downhill (HD and BBL), consistently with experimental interpretation. Figure derived with permission from Lindorff-Larsen et al. [177].

Despite the impressive advances in experimental methods described above, atomistic MD simulations are possibly the only practical approach to resolve folding transitions of individual molecules with the time and structural resolution required to derive mechanistic information. Fast folding has stimulated the development and benchmarking of various approaches based on MD simulations [59,63,138,173–176]. Recently, Shaw et al. [62] used their Anton supercomputer to reach the near-microsecond simulation times required to watch fast-folding proteins fold and unfold multiple times and simulated 12 experimentally studied fast-folding proteins with diverse topologies [177]. The simulations folded most of these proteins into their native structure multiple times and with rates similar to those determined experimentally [177]. A key result was the confirmation that fast-folding proteins cross very small barriers, and that some of them truly fold in the one-state downhill fashion proposed by Muñoz and co-workers [130,139] (Figure 6). In the simulations, collapse and secondary structure occurred together to form a compact form in which a native-like topology was stabilized by a small subset of key long-range native contacts. Detailed analysis of the trajectories indicated that the productive folding pathways (order of structural events) are relatively homogeneous, although their heterogeneity increased for larger proteins, particularly those containing β-structures [177].

The possibility of simulating multiple folding–unfolding transitions in single trajectories offers very exciting possibilities to investigate folding mechanisms in detail. The simulations provide the extreme resolution that experiments could never achieve, but still use approximate force fields to describe protein energetics and dynamics. Increasingly sophisticated experiments, such as those described in the present review, provide the critical benchmarks for further refinement of simulations in a perfect symbiosis. First steps in this direction have been taken by combining experiments and MD simulations to design mutations that speed up folding of WW domains [125], to investigate the folding interaction network and mechanisms of the protein gpW [162], and to investigate the barrier-crossing process of the designed protein α_3_D [172].

## BIOLOGICAL ROLES AND APPLICATIONS OF FAST FOLDING

A related area of interest focuses on the structural and functional analysis of IDPs [178], especially after the realization that IDPs amount to a very large fraction of the proteome [179]. These proteins exhibit structural disorder in native conditions and folding coupled to binding via complex mechanisms [180]. Experimental studies have reported IDPs that bind to their partners through either induced fit or conformational selection mechanisms [181]. Some IDPs bind to multiple partners that are structurally diverse [182], a feature that allows them to moonlight [183] or produce sophisticated allosteric effects [184].

**Figure 7:**
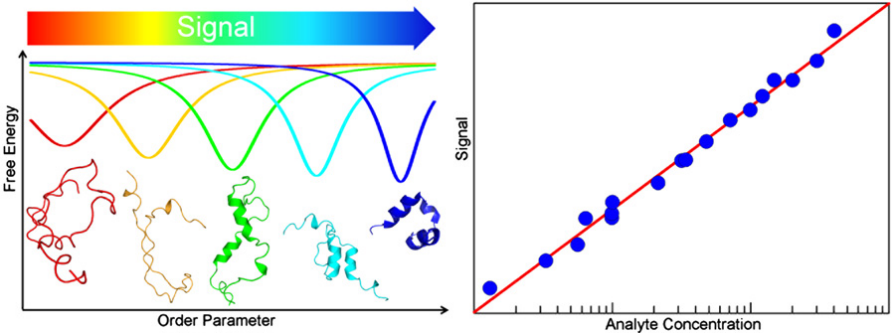
Biological and technological roles of conformational rheostats Left: a conformational rheostat is based on a downhill folding domain near its denaturation midpoint. The partially folded conformational ensemble of this domain can gradually become more or less structured by coupling folding to a signal such as binding to one or various partners. Right: the conformational rheostat concept has been used to implement folding coupled to binding biosensors that exhibit a much broader dynamic response than conventional conformational switches

It turns out that IDPs and fast-folding proteins, especially one-state downhill folders, are closely interconnected. It has been noticed recently that folding rate, stability and co-operativity are intimately coupled so proteins that fold fast also unfold fast, are marginally stable and are minimally co-operative [156]. In fact, the stability of domains identified as downhill folders seems to be poised towards exhibiting partial disorder under physiological conditions. This trend has been observed by investigating homologous fast-folding domains from meso-, thermo- and hyper-thermophilic organisms in which the denaturation temperature of the domain tracks the living temperature of the organism [185]. So-called IDPs, on the other hand, have significant residual structure, as shown by NMR [186] and single-molecule fluorescence [187] experiments, and form stable native structures under slightly favourable thermodynamic conditions [188,189]. The ability of IDPs to be both partially disordered and poised to fold up with slight thermodynamic input seems to be a simple manifestation of their one-state downhill folding character [185]. Such folding characteristics enable their operation as conformational rheostats, that is molecular devices capable of producing analogical signals in response to binary stimuli such as binding to specific partners [130,156,185] (Figure 7, left). In this light, the functional complexity and multiple binding modes reported on IDPs could be explained as emerging from the coupling between binding and downhill folding. There is mounting evidence that the complex binding modes observed on IDPs involve gradual conformational changes rather than binary transitions.

For example, NCBD (nuclear co-activator-binding domain) has been classified as an IDP [188,190] that binds multiple partners by folding into different conformers [188,191,192]. At the same time, NCBD is capable of folding into a three-helix bundle structure in the presence of stabilizing agents, and it does so following a gradual process (one-state downhill) according to the multivariate analysis of multi-probe experimental data and computer simulations [193]. Another interesting case is the PSBDs (peripheral subunit-binding domains) from several multienzymatic complexes, such as the pyruvate and 2-oxoglutarate dehydrogenases [194], which include the first identified examples of one-state downhill folding [130]. In these multienzymes, the catalytic process involves four steps catalysed by three subunits (E1, E2 and E3) that form a dynamic macromolecular complex, one that is fully controlled by the interactions between the PSBD from the E2 subunit and the E1 and E3 subunits [194]. E1 and E3 are structurally very different, yet the small PSBD (<50 residues) is capable of binding to both exclusively, with high affinity (*K*_d_ of 0.33 and 0.58 nM) and 1:1 stoichiometry [195]. Moreover, the crystallographic structures of E1 and E3 bound to the PSBD show very superficial binding interfaces and extremely high mobility in the PSBD region (*B*-factors up to 80 Å^2^; 1 Å=0.1 nm) [196,197], suggesting that PSBD is largely disordered when bound. Certain DNA-binding proteins, such as homeodomains, are also likely candidates for conformational rheostats. These domains face the enormous challenge of finding a short target sequence within the enormous pool of potential binding sites provided by genomic DNA. To solve this problem, they exhibit specific and non-specific DNA binding [198] that they aptly combine to slide (1D diffusion) and hop (2D diffusion) over DNA resulting in search speed-ups of at least ∼100-fold relative to a standard 3D diffusion-controlled processes [199]. DNA sliding has been studied theoretically [199], computationally [200] and experimentally using single-molecule methods [201] and paramagnetic relaxation enhancement NMR [202]. The molecular mechanism by which DNA-binding proteins implement these two binding modes remains largely unclear, however. But we now know that DNA-binding domains are marginally co-operative fast folders [155], exhibit partial disorder when unbound to DNA [203], and seem to fold via a downhill mechanism [171]. These properties suggest a molecular rheostat in which the conformational motions of a partially unfolded domain are exploited to counterbalance DNA processivity and sliding speed during non-specific binding, and ensure quick locking into the target sequence. These domains also bind non-specifically to DNA in a manner that seems to be DNA-sequence-dependent [198], which further suggests a homing-to-target mechanism mediated by conformational selection.

Finally, conformational rheostats also offer very attractive possibilities for technological applications. A first effort in this direction has targeted the design of high-performance biosensors based on gradual conformational changes coupled to proton binding [204]. The authors of that work exploited the natural properties of the BBL domain in terms of folding [130] and proton binding [205] to engineer a pH ionic strength sensor with linear response over five orders of magnitude in analyte concentration, instead of the two orders that are inherent to conformational switches (Figure 7, right). Moreover, these sensors exhibited ultrafast response thanks to the microsecond folding kinetics of BBL and the gradual coupling between folding and binding [204].

## Abbreviations

DSC: differential scanning calorimetry
IDP: intrinsically disordered protein
NCBD: nuclear co-activator-binding domain
PSBD: peripheral subunit-binding domain
RD-NMR: relaxation dispersion NMR
